# Somatic awareness in the clinical care of patients with body distress symptoms

**DOI:** 10.1186/1751-0759-2-6

**Published:** 2008-02-21

**Authors:** Donald Bakal, Patrick Coll, Jeffrey Schaefer

**Affiliations:** 1 Department of Medicine, University of Calgary, Calgary, Canada; 2 Clinic for Mind/Body Medicine, Calgary Health Region, Calgary, Canada; 3 Department of Psychiatry, University of Calgary, Calgary, Canada

## Abstract

The purpose of this paper is to provide primary care physicians and medical specialists with an experiential psychosomatic framework for understanding patients with body distress symptoms. The framework relies on somatic awareness, a normal part of consciousness, to resolve the dualism inherent in conventional multidisciplinary approaches. Somatic awareness represents a guiding healing heuristic which acknowledges the validity of the patient's physical symptoms and uses body sensations to identify the psychological, physiological, and social factors needed for symptom self-regulation. The experiential approach is based on psychobiologic concepts which include bodily distress disorder, central sensitization, dysfunctional breathing, and contextual nature of mood.

## Background

Symptoms of somatic or body distress (BD), more widely known as medically unexplained symptoms (MUS) or functional somatic syndromes, are characterized by patterns of persistent physical complaints for which adequate examination does not reveal specific pathology [1]. We prefer the term BD as it directs attention to psychobiologic processes that underlie the symptoms and that need to be recognized and self-managed by the patient. The term BD mirrors patients' symptom experiences and avoids the psychocentric "all in the head" implication often associated with MUS [2]. Primary and specialist care are where the majority of BD patients are managed and clinicians are often challenged in determining the right approach to effective treatment. Although physicians want to be able to treat BD patients, they believe that these patients are difficult to manage and that selecting the right approach to treatment with any particular patient is often "a shot in the dark" [3]. An element of randomness is discernible in treatment guidelines which often recommend when conventional treatments fail that practitioners consider a variety of alternative therapies ranging from biofeedback to naturopathy.

Patient dissatisfaction with the medical care they have received is well-documented. Patients often feel invalidated and treated as if they were "medical orphans." They complain that their physical symptoms are not taken seriously and that their physicians do not listen and only attempt to provide relief through medication. The same patients reject suggestions that their symptoms might have a psychosomatic origin. For instance, a chronic fatigue patient is quoted saying "It (the illness) hit at a time when I couldn't have been more fulfilled. So at no time must anyone dare tell me it is all in the mind" [4]. These patients harbour the hope that some day biomedical science will identify an organic cause, and fix "it" once and for all.

In natural science the difficulty of explaining how the mind putatively influences the body is known as the explanatory gap. Nowhere in clinical medicine is the gap a greater problem than in the management of BD conditions such as irritable bowel syndrome, fibromyalgia, chronic fatigue, non-cardiac chest pain, and chronic headache. There is a huge gap between what physicians think about patients with symptoms of BD and what patients expect from their physician, often resulting in stressful consultations [5]. The gap continues to interfere with efforts to explain these symptoms in a fashion that is acceptable to both physicians and patients [6].

Mind-body separation or dualism is implicit in psychosocial treatment models for BD. Reattribution is a structured consultation administered by physicians which aims to provide a psychological explanation for physical symptoms. The goal of treatment is to shift the patient from a biological to a psychological understanding of his/her symptoms. An example from reattribution therapy would involve telling the patient that his/her symptoms are the result of a "hormonal imbalance" which itself is the result of perfectionism and deficient self-care. The explanation does not resonate with patients. They find the explanation too psychological and removed from actual symptom experiences. Reattribution has been found to be effective in improving the doctor-patient relationship but not effective in improving patients' perceived overall health [7].

Cognitive-behavior therapy represents the widely recommended non-drug treatment for all forms of BD [8]. The successfulness of this approach has been overstated, especially in terms of providing long term relief of symptoms. The model assumes that a patient is interpreting benign or 'normal' physical sensations catastrophically as indicative of something dangerous. The approach emphasizes the psychologic distress rather than the BD. The cognitive model is limited, in the same way as reattribution, by a failure to address the psychobiology of the patient's symptom experience. This may also partly explain why physicians are reluctant to adopt psychosocial approaches within their practice [9].

## The concept of bodily distress disorder

Reviews of functional somatic syndromes conclude that patients with diagnostic symptom criteria from one syndrome (irritable bowel), are inclined to exhibit symptoms from one or more of the other syndromes (fibromyalgia, chronic fatigue) [10,11]. For instance, the tender point sensitivity criteria used by rheumatologists to diagnose fibromyalgia are not specific to fibromyalgia patients [12]. Functional syndromes may be an artefact of the medical specialist's tendency to focus on symptoms pertinent to his/her specialty.

The concept of bodily distress disorder (BDD) has been proposed by Fink, Toft, Hansen, Ørnbøl and Olesen [13] to unite symptom overlap along a single severity dimension. The researchers interviewed 978 patients who had been investigated in neurology, internal medicine, and primary care for the presence of functional symptoms. The initial aim of the study was to empirically examine the validity of the most common functional syndromes described in clinical practice. A factor analysis of 62 functional symptoms found limited support for the presence of cardiopulmonary, gastrointestinal, and musculoskeletal/pain factors. The three-factor model explained only 36.9% of the variance and the identified factors were intercorrelated, meaning that patients with symptoms from one group were inclined to report symptoms from one of the other symptom groups. A further latent class analysis revealed that the patients could be more accurately classified into three classes defined by the total number of symptoms they presented from any of the three symptom groups.

In order to successfully classify patients within a BDD model, Fink et al. [13] relied on five key symptoms which were observed to be common across the major clinical syndromes: headache, dizziness, memory impairment, concentration difficulties and fatigue. Of relevance to the present thesis is the observation that these symptoms are also central to definitions of hyperventilation and can have their origins in dysfunctional breathing patterns. Moreover, these symptoms are at the top of the list of BD symptoms most frequently observed in primary care settings [14]. We have observed that dysfunctional breathing in the form of breath holding, tightness in the chest, and difficulty breathing deeply, is reported by the majority of BD patients. Dysfunctional breathing may serve as a proxy for an increased sensitivity to physical, sensory, cognitive and affective symptoms.

## Depression and sadness in context

Somatic symptoms in the absence of detectable organic pathology are often assumed to represent "masked" depression and are often treated with antidepressants. There is a high incidence of depression and other mood symptoms in BD patients. Fink et al. [13] found that BDD was associated with symptoms of depression and anxiety. The strongest association was found with unspecific emotional distress, defined by symptoms of worrying, sensitivity to noise, irritability, feeling nervous, general muscular tension, and restlessness. Review articles conclude that depression is responsible for most MUS seen in primary care [15]. However, BD patients do not accept having their physical symptoms categorized as depression. There is little support for the usefulness of antidepressants in treating specific syndromes or BD symptoms in general. Prolonged use of antidepressant medications contributes to drug dependence, illness beliefs, disempowerment, and often exacerbates the symptoms that are being treated [16].

Horwitz and Wakefield [17] argue that contemporary psychiatry confuses normal sadness with depressive mental disorder and overuses depression treatments. Normal sadness can be associated with symptoms of depression including depressed mood, loss of interest in usual activities, insomnia, inability to concentrate and other Diagnostic and Statistical Manual of Mental Disorders (DSM) classified depression symptoms. Sadness, however, is a normal reaction to particular kinds of losses and not the result of a dysfunction in an internal brain mechanism. Sadness is a psychobiologic condition with somatic and psychic symptoms which occur in the context of the patient's emotional and interpersonal life.

Identifying the context of reported depressive and other symptoms of emotional distress by BD patients is critical for effective management. An example is provided by a 27-year old married woman with functional abdominal cramps and bloating who described herself as being "depressed my entire life." She developed abdominal pain at 9 years of age and began "sucking in my gut because of the pain and sucking up my feelings because of my mother." At 10 years of age she lost her mother to lung cancer. Following the death of her mother, her father began an affair with her mother's sister. Her father died from lung cancer two years later. The patient described being mocked throughout adolescence as the "fat kid." At 12 years of age she was diagnosed with fibromyalgia. She is currently 30–40 pounds overweight and smokes a package of cigarettes a day. She was recently married and after one month her husband had an affair and requested a divorce. The couple is seeing a marital counsellor but she remains distrustful, pessimistic and in chronic pain. Her husband was prescribed an antidepressant to control anger outbursts and she fears that any improvement is the "result of the drug and not him" and not likely to last. By experiencing her body in the context of emotions, the patient was able to begin to understand the connection between past and present traumatic experiences and the 18-year history of abdominal cramps and pain. It is important to emphasize that somatic awareness is a guide and not a cure. There is no quick fix to treating patients with a long history of depression symptoms and BD. We do not believe, however, that antidepressant treatment is the answer.

## Tacit knowing and somatic awareness

It is widely recognized that medicine needs to shift from an exclusively biomedical model to a biopsychosocial model in the understanding of health and illness. Physicians generally express strong appreciation for the importance of psychosomatic factors in treating patients [18]. However, they also believe that there are a number of training and conceptual barriers to adopting a biopsychosocial approach in their practice [19]. For example, they have been trained to view conditions as purely biological or psychological in etiology and treatment. They also question their ability to evaluate the evidence offered in support of holistic techniques such as meditation, relaxation, and guided imagery.

A proposal has been made that medicine move beyond evidence-based rules and technologies to include tacit knowing as a legitimate form of knowledge, understanding and judgment in clinical decision-making. Tacit knowing refers to the "taken-for-granted knowledge" at the periphery of attention that allows people to understand the world and discern meaning in it [20].

"When a physician listens to a patient describe his or her symptoms, for example, the physician pays explicit attention to the patient's story. The physician is simultaneously aware of the patient's tone of voice, facial expressions, and choice of words, but is aware of them in a qualitatively different way. The physician appreciates these subsidiary phenomena tacitly; that is, only to the extent that they provide a background that makes the physician's explicit knowledge of the patient's story intelligible and meaningful." (p. 294)

The paradigm for tacit knowing was developed by the physician-philosopher Michael Polanyi [21] and is heavily based on body experience. Tacit knowing is also at the basis of humanistic understanding of patients and their distress. To achieve an integration of explicit and subsidiary knowing of BD patients, clinicians can watch for evidence of BD via shallow and irregular breathing, jaw tightness, forehead furrowing, rapid speech, neck and shoulder rigidity, fist clenching, leg bracing and so forth. Patients will then be able to begin connecting body events and symptoms to thoughts, feelings and personal circumstance.

We propose that psychosomatic approaches can be better integrated into clinical practice by the inclusion of somatic awareness as a form of tacit knowing. Somatic awareness involves directing a patient's attention to interoceptive or body experience and associated feelings for the purpose of self-healing and achieving health [22]. The use of this healing heuristic in primary care would advance the understanding of how the body self-heals. Somatic awareness serves as a powerful clinical tool to facilitate communication and humanistic care between physician and patient.

There are conceptual and practical advantages to using an experiential body heuristic in patient care. These include: 1. The utilization of a normal conscious experience that is readily recognized by patients. 2. The experience of a subjective state that is complementary to body states associated with healing and wellness. 3. The adoption of a consciousness concept that is consistent with recent theoretical formulations in neurobiology. 4. The use of an experiential guide that networks the biological, psychological and social variables unique to each patient.

With somatic awareness, we are not advocating that physicians begin offering holistic treatment modalities in their practice but rather adopt a shared conceptual framework of health and healing with their patients. Somatic awareness is an experiential guide or meta-skill rather than a treatment technique. Physician and patient can decide together what modality treatments and resources available in the community might strengthen this skill and the patient's self-healing.

There are also theoretical reasons for making somatic awareness central to the healing process. Modern neurobiology has adopted a broad approach to interoception which extends beyond the viscera to include sensations related to the physiological condition of all organs and structures of the body – including muscles, joints, teeth, skin and connective tissue [23]. Also there is recognition that the subjective process of healing is intricately related to same brain regions that are involved in mapping of internal bodily states. The mind, according to Damasio [24], is not only 'embrained' but also 'embodied."

## Incorporation of somatic awareness into medical practice

Somatic awareness can serve to network the physiological, psychological and contextual variables unique to each patient that need to change. The psychobiologic components of BD within each patient can be pinpointed with clinical precision and a collaborative treatment plan developed. Somatic awareness is used to guide changes in disease/illness beliefs, body schema, coping styles, interpersonal dynamics, sleep, and medication dependence. Psychological influences are described as psychobiologic in nature rather than mental phenomena. It is much easier for the patient to accept, experience and alter the impact of previous trauma, personality, stress and emotions if he/she views the body as the mediator of these experiences. The specific BD domains which need to be identified are listed in Table 1. Positive answers to the majority of these questions can be expected. However the experiential context of the patient responses is highly individualized making a cookbook treatment approach less than desirable.

**Table 1:** BD interview questions and observations

1.	Does the patient ignore his/her body symptom(s) and try to "push on?"
2.	Does the patient exhibit perfectionism and/or Type A attributes?
3.	Does the patient suppress or "stuff" negative feelings?
4.	Did the patient experience verbal, physical and/or sexual abuse as a child?
5.	Is the patient's sleep non-restorative?
6.	Is the patient fearful of functioning without medications?
7.	Is the patient presently experiencing marital/interpersonal problems?
8.	Does the patient exhibit elevated shoulders, facial tension or body guarding?
9.	Does the patient report teeth clenching/grinding?
10.	Does the patient exhibit dysfunctional breathing?
11.	Does the patient breathe into the chest when instructed to take a "relaxed" breath?

There are several themes that need to be integrated into an individualized patient care plan:

### 1. Shift from disease to BD schema

Patients with BD symptoms often have strong disease-related beliefs and concerns in association with their syndrome label. These concerns can be addressed throughout the treatment process by having the patient understand his/her condition as a form of BD. BD is a psychobiologic construct and has an explanatory advantage over the psychological and psychosocial explanations associated with reattribution models [7]. Introducing psychological and psychosocial issues as a causal explanation is countered by giving the impression to patients that their symptoms are not regarded as legitimate. The notion of BD intuitively validates the origins and physical reality of the patients' experiences with the symptoms. The concept also helps patients appreciate the interface of physical symptoms and emotional and physiological reactions. Through somatic awareness, patients and practitioners are able to collaborate in the development of an experiential self-management treatment plan.

The concept of body schema is used to organize the psychobiologic changes which need to occur for effective symptom self-management. Body schema is a system of sensory-motor functions that operate in a *close to automatic *fashion at a preconscious level [25]. Awareness of body sensation is generally pushed aside by some cognitive, emotional or situational issue. The reactions of the body are usually on automatic pilot – until a symptom develops and demands attention. Tacit understanding of patient body language is very useful in guiding patients towards greater somatic awareness. Having the patient demonstrate his/her understanding of an effortless or relaxed breath is a quick way to assess how the patient's body schema interprets and performs the request. Learning to modify body schema takes practice and guidance and cannot be expected to happen in a single session. Patients need to develop an awareness of the link between their body reactions and the emotional/situational context and begin altering their body's habitual way of responding. Effortless (relaxed, abdominal) breathing can be readily demonstrated by having the patient place his/her hands on the abdomen and chest and breathe in a relaxed manner. The objective of such a demonstration is not breathing re-training but having the patient experience a moment of physical relaxation and of relaxation and well-being.

### 2. Self-soothing and awareness of feelings

Paradoxically, BD patients are known for ignoring their body outside of symptom experiences. This strategy denies access to contextual warning sensations that can be used to prevent symptom onset. Lack of awareness of breathing is a common example – patients may literally stop breathing in demanding situations and only later experience dizziness and difficulty concentrating. It is common for patients, especially those with chronic fatigue and fibromyalgia, to harbour a determination "not to give in" – a strategy which paradoxically makes the illness worse. Patients will attempt to "ignore" the symptoms or "push on" which eventually leads to further frustration, hopelessness, and perceived functional limitation.

The origins of body avoidance can be traced to emotional avoidance. Problems with early attachment are very common. BD patients have often experienced emotional, physical, and/or sexual abuse. Patients should always be queried regarding emotional closeness with parents as well as an abuse history. Patients who have experienced adverse childhood events believe that they have "moved on." They fail to appreciate how embodied traumatic experience continues to influence their somatic symptoms. Also, the notion of body self-soothing is associated with guilt over wasting time, giving up, and fear of losing control. Traumatised patients faced with overwhelming emotions lose their ability to use emotions as guides for effective action, resulting in an inability to identify the meaning of physical sensations and muscle activation. New approaches to trauma treatment are recommending that victims receive treatment in the form of body-oriented therapies that increase the capacity for attending to inner sensations, perceptions and feelings [26].

BD patients who have experienced early trauma are often oblivious of their body sensations. Through somatic awareness, they are dismayed to discover that their body remains hypervigilant and continues to influence and be influenced by current feelings and interpersonal interactions. The body is indeed the "unconscious mind." A patient with non-cardiac chest pain reported an emotionally abusive childhood with an alcoholic father. Some 50 years later, she continued to experience chest tightness and/or pain in the presence of a controlling husband as well as in other uncomfortable social situations. Instructions in relaxation, breathing awareness, and suggestions to improve spousal communications led to significant reductions in the tightness and pain. Body avoidance is also characteristic of their current approach to emotional and interpersonal life. They dislike emotional confrontation and they can be living with controlling parents, partners and children. With irritable bowel patients we often hear that they prefer to "stuff" their feelings towards family members. They generally do not connect or may even deny the relationship between bowel symptoms and family conflicts. With the development of somatic awareness, they learn to recognize that their physical symptoms have a connection to feelings and interpersonal situations.

### 3. Medication withdrawal and sleep

Patients do not benefit from somatic awareness therapy unless medication withdrawal is part of the treatment process. These patients have spent a lifetime ignoring their emotional and body experience. Medicating an emerging sense of emotional and body awareness with psychotropic, analgesic and anticonvulsant medications impedes their ability to utilize interoceptive healing information from the body. Patients who have been relying on medications have strong fears that they will relapse or worsen without medication, even if the medication is ineffective. The chemical imbalance theory of depression when given to patients is notorious for exacerbating this fear.

Non-restorative sleep is a defining characteristic of BDD. The "fear of fear" of not sleeping is frequent. Sleep hygiene is recommended but is not sufficient to alter the worry and somatic anxiety and/or pain which these patients experience in bed. They believe that if they can achieve sleep with the aid of hypnotics, they will be able to cope, even if they are not coping. They need to understand that a drug-induced sleep will not alter the BD cycle. A patient's body reactions and accompanying fantasies during drug-induced sleep reflect daytime themes and coping styles. A body that is guarded throughout the day will not necessarily "let go" and relax during the night, leaving the patient exhausted in the morning. We encourage patients to adopt somatic awareness through body scanning, self-soothing thoughts and feelings, and effortless breathing prior to sleep onset. Patients may initially exhibit strong fears of trying to sleep without medication and need support and encouragement. We advise that rest is as good as sleep – not entirely true, but if practiced, sleep will come.

## Summary

In summary, a patient-centered and experiential framework is proposed for helping physicians navigate the complex management of the patient with BD symptoms. The focus on somatic awareness avoids the physical-psychological dualism inherent in conventional models. It also provides a non-confrontational framework that acknowledges both the validity of the patient's physical symptoms and the identification of psychological and social factors needed for the healing process. Therapeutic progress takes time as patients need to identify individual and contextual variables within themselves which can be used to self-manage their illness. Somatic awareness can also be used by physician and patient to determine which allied health professionals and community-based healing resources might best strengthen the patient's self-management skills and goal of wellness. Somatic awareness serves to guide the listening, exploration and validation process that is required for effective patient care. The concept recognizes the existence of inner healing processes complementary to biomedicine and provides physician and patient a treatment focus on healing rather than disease.

## Abbreviations

BD: body distress; BDD: bodily distress disorder; DSM: Diagnostic and Statistical Manual of Mental Disorders; MUS: medically unexplained symptoms

## Competing interests

The author(s) declare that they have no competing interests.

## Authors' contributions

DB wrote the initial draft of the paper and PC and JS contributed to the revisions. All authors read and approved the final submission.
